# Chlorination of Aromatic Amino Acids: Elucidating Disinfection Byproducts, Reaction Kinetics, and Influence Factors

**DOI:** 10.3390/molecules29081879

**Published:** 2024-04-20

**Authors:** Zhenyi Chen, Bangyu Chen, Hui Shen, Xunlin Li, Chen Zhou, Guangcai Ma, Xiaoxuan Wei, Xueyu Wang, Haiying Yu

**Affiliations:** College of Geography and Environmental Sciences, Zhejiang Normal University, Yingbin Avenue 688, Jinhua 321004, China; mn415czy@163.com (Z.C.); chenbangyuu@163.com (B.C.); shenhui182@163.com (H.S.); 20222025@zjnu.edu.cn (X.L.); zhouchen_02@163.com (C.Z.); xxwei@zjnu.edu.cn (X.W.); xywang@zjnu.edu.cn (X.W.)

**Keywords:** chlorination, aromatic amino acids, disinfection byproducts, reaction kinetics, chlorine dosage, pH value

## Abstract

In the face of ongoing water pollution challenges, the intricate interplay between dissolved organic matter and disinfectants like chlorine gives rise to potentially harmful disinfection byproducts (DBPs) during water treatment. The exploration of DBP formation originating from amino acids (AA) is a critical focus of global research. Aromatic DBPs, in particular, have garnered considerable attention due to their markedly higher toxicity compared to their aliphatic counterparts. This work seeks to advance the understanding of DBP formation by investigating chlorination disinfection and kinetics using tyrosine (Tyr), phenylalanine (Phe), and tryptophan (Trp) as precursors. Via rigorous experiments, a total of 15 distinct DBPs with accurate molecular structures were successfully identified. The chlorination of all three AAs yielded highly toxic chlorophenylacetonitriles (CPANs), and the disinfectant dosage and pH value of the reaction system potentially influence chlorination kinetics. Notably, Phe exhibited the highest degradation rate compared to Tyr and Trp, at both the C_AA_:C_HOCl_ ratio of within 1:2 and a wide pH range (6.0 to 9.0). Additionally, a neutral pH environment triggered the maximal reaction rates of the three AAs, while an acidic condition may reduce their reactivity. Overall, this study aims to augment the DBP database and foster a deeper comprehension of the DBP formation and relevant kinetics underlying the chlorination of aromatic AAs.

## 1. Introduction

The disinfection of drinking water is crucial to ensure its safety and to eliminate pathogenic microorganisms [1,2]. Various methods such as chlorine, chloramine, chlorine dioxide, ozone, and ultraviolet disinfection are employed for this purpose. Among these, chlorine disinfection stands out globally for its remarkable bactericidal effect and cost-effectiveness [3]. It is extensively utilized, with over 94.5% of water treatment facilities in the United States and over 99.5% in China relying on chlorine disinfection [4]. However, while disinfectants effectively inactivate or eliminate harmful microorganisms, their strong oxidizing properties can trigger unavoidable reactions with natural organic matters (NOMs) and bromide and iodide compounds present in water, leading to the formation of disinfection byproducts (DBPs) [5] that pose health risks. For instance, during chlorine disinfection of drinking water, trihalomethanes (THMs) and haloacetic acids (HAAs) [6,7] are produced, which are known to be carcinogenic and reproductively toxic [8,9]. Notably, the chlorination reactions of wastewater also lead to the formation of a substantial number of DBPs, which are generally more toxic than the parent compounds and even more resistant to subsequent degradation. These byproducts are present in the water released into the environment, where they can accumulate in surface water, ultimately contributing to the overall detection of total DBPs in drinking water [10,11,12].

In recent years, aromatic DBPs have garnered increasing attention as a particularly hazardous class of DBPs [13,14,15,16]. In vitro cytotoxicity experiments have shown that aromatic DBPs typically exhibit toxicity levels several orders of magnitude higher than aliphatic DBPs [17,18,19]. Notably, chlorophenylacetonitriles (CPANs) have emerged as stable aromatic nitrogen-containing DBPs with significant toxicity [20]. The *LC*_50_ values of 2-chlorophenylacetonitrile (2-CPAN) and 3,4-dichlorophenylacetonitrile (3,4-DCPAN) were 133 and 83 μmol/L, respectively, significantly lower than those of their aliphatic counterparts, chloroacetonitrile, and dichloroacetonitrile (436 and 260 μmol/L, respectively) [21]. Despite their toxicity, the formation mechanisms and major precursors of CPANs remain elusive.

Amino acids (AAs), ubiquitous components of NOM in aquatic environments, enter the drinking water preparation process through the water supply system. They present at concentrations ranging from 20 to 10,000 μg/L in surface waters and represent a significant fraction of dissolved organic carbon and organic nitrogen [22,23]. During chlorine disinfection, AAs undergo conversion into various carbonaceous and nitrogenous DBPs [24]. Removing free AAs, especially aromatic ones, poses challenges to conventional water treatment methods. These aromatic AAs can serve as primary precursors for highly toxic DBPs [25]; for instance, tyrosine (Tyr) and tryptophan (Trp) can yield trihalomethanes [26], while phenylalanine (Phe) may generate CPANs and phenylacetaldehyde during water disinfection process [27,28]. Due to their structural diversity, different AAs ultimately lead to various types of DBPs, necessitating a thorough understanding of the chlorination of AAs and the resulting DBP profiles.

Furthermore, elucidating the kinetics of reactions between precursors and disinfectants sheds light on disinfection reaction rates, mechanisms, and the impacts of various factors on disinfection efficiency, consequently providing a theoretical basis for optimizing the disinfection process [29]. Currently, extensive research is being conducted on the kinetics of disinfection reactions in drinking water. In addition to the traditional focus on microbial disinfection reactions, considerable attention is also being paid to the kinetics of disinfection reactions for various NOMs and synthetic chemicals present in water. For instance, the chloramination of inosine [30] and the chlorination of N-phenyl-N′-(1,3-dimethyl butyl)-p-phenylenediamine-quinone (6PPDQ) [31] and prometryn [32] can efficiently occur, yielding various DBPs, with their reactions following pseudo-first-order kinetics. While the kinetic studies of AA chlorination in drinking water are mostly related to their reaction mode, it has been demonstrated that the reactions between Tyr and Trp with ClO_2_ follow second-order kinetic reactions with the rate constants ranging from 10^2^ to 10^7^ M^−1^s^−1^ [33]. Because of the rapid chlorination reaction of AAs, relatively few detailed studies have been conducted on the various factors that influence the kinetics of their chlorination processes.

Building upon existing research, this study undertakes chlorination experiments on three representative aromatic AAs, including tyrosine, phenylalanine, and tryptophan, with the aim of identifying the molecular structures of potential DBPs to enrich the existing DBPs database. Concurrently, disinfection kinetics experiments are performed to investigate the impacts of various factors, including chlorine concentration and pH, on reaction rates. Anticipated results are poised to offer valuable insights into a deeper comprehension of the mechanism underlying DBP generation, particularly focusing on CPANs originating from aromatic AAs during water disinfection. By shedding light on these processes, this research endeavors to contribute significantly to the understanding and management of DBP risks in water treatment.

## 2. Results and Discussion

### 2.1. Identification of the Chlorination DBPs of Three Aromatic AAs

#### 2.1.1. DBPs of Tyr Chlorination

**Figure 1:**
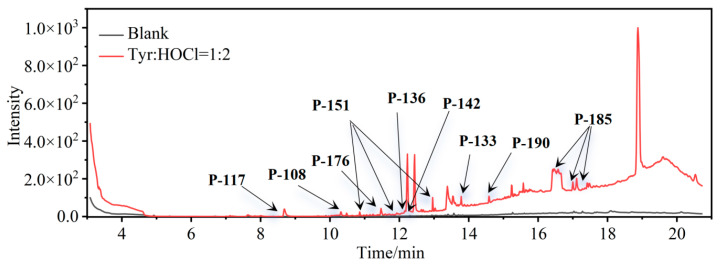
Total ion chromatogram of Tyr chlorination (pH = 7.0, 25 °C, Tyr:HOCl = 1:2, 24 h).

During chlorine disinfection, the chemical transformation of Tyr entails substitution, decarboxylation, and hydrolysis, resulting in the formation of aldehydes, nitriles, and Cl-substituted DBPs. The chromatographic analysis depicted in Figure 1 illustrates the comprehensive profile of Tyr chlorination, conducted at a concentration ratio of 1:2 (C_Tyr_:C_HOCl_) for 24 h under conditions of pH = 7.0 and T = 25 °C, with precautions taken to avoid exposure to light. A comparative assessment with a blank sample (represented by the black line in Figure 1) was performed, followed by a rigorous spectral analysis employing the National Institute of Standards and Technology (NIST) Mass Spectral Library and associated Search Program to elucidate the structural attributes of the resultant compounds.

Via meticulous comparison and systematic search methodologies, the investigation revealed the emergence of nine DBPs originating from Tyr chlorination under the specified conditions, including 4-hydroxytoluene (P-108), 4-propylphenol (P-136), *p*-hydroxyphenylacetonitrile (P-133), 2,6-dichloro-4-methylphenol (P-176), 2,6-dichloro-4-methylphenol (P-142), phenylacetonitrile (P-117, PAN), 2,6-dichloro-4-ethylphenol (P-190), monochlorophenylacetonitrile (P-151, mono-CPAN), and dichlorophenylacetonitrile (P-185, di-CPAN), with detailed information provided in Table 1. Particularly noteworthy is the generation of two CPANs (P-151 and P-185), highlighting Tyr’s susceptibility to undergo chlorination-induced transformation to yield highly toxic aromatic DBPs. Therefore, recognizing Tyr as a potential precursor for such CPANs underscores its significance in the context of water treatment protocols.

In the investigation of structural characteristics of DBPs derived from Tyr, successive examinations were conducted on reaction systems featuring various molar ratios of Tyr to active chlorine (Tyr:HOCl). The resulting findings are presented in Table 2. Analysis revealed distinct product profiles corresponding to different chlorine concentration levels applied to Tyr, with a proliferation of DBP variants, observed as the concentration ratio of active chlorine surpassed 2:1. Conversely, a reduction in the diversity of DBPs occurred when the concentration of active chlorine fell, with products P108, P-176, and P-190 exclusively emerging under conditions of heightened available chlorine concentrations. Notably, the appearance of P-185 (di-CPAN) occurred subsequent to achieving a 1:1 ratio between Tyr and available chlorine, indicating the sequential formation of di-CPAN following mono-CPAN in the reaction system. Furthermore, the generation of P-151 (mono-CPAN) was observed even under lower available chlorine concentration, underscoring the propensity for CPAN production despite reduced chlorine availability.

#### 2.1.2. DBPs of Phe Chlorination

**Figure 2:**
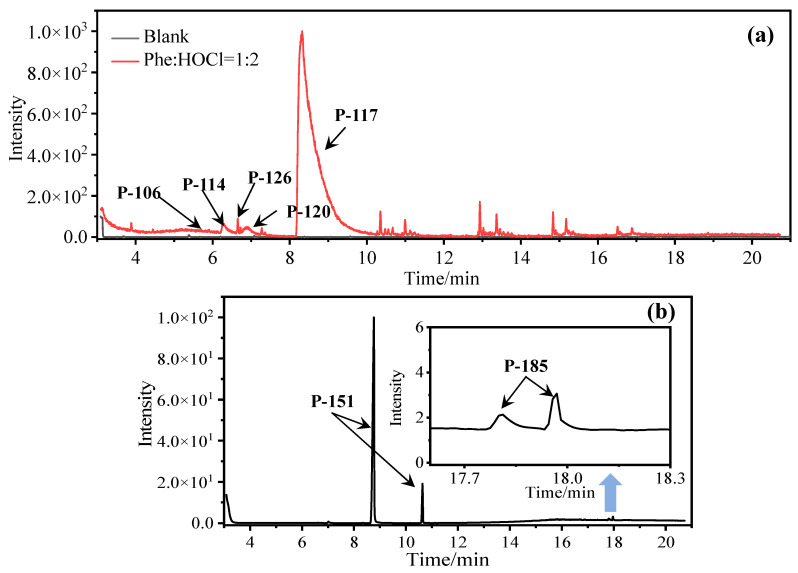
(**a**) Total ion chromatogram of Phe chlorination (pH = 7.0, 25 °C, Phe:HOCl = 1:2, 24 h) and (**b**) Selective ion monitoring chromatogram of generated CPANs under the same conditions.

In comparison to Tyr, the chlorination of Phe exhibited reduced complexity of resulting DBPs due to the absence of the hydroxyl group in the benzene ring. This process did not generate the hydroxylated products but unexpectedly led to the formation of aldehydes. Chromatographic representations elucidating the obtained products of Phe chlorination are depicted in Figure 2, while detailed specifics regarding each product are given in Table 1. Throughout the chlorination process of Phe, seven distinct DBPs emerged, including the three identical phenylacetonitrile derivatives (P-117, P-151, and P-185) observed in the case of Tyr. The other four DBPs are identified as benzaldehyde (P-106), phenylacetaldehyde (P-120), chlorobenzene (P-114), and benzyl chloride (P-126). Furthermore, the selective ion monitoring chromatogram depicted in Figure 2b effectively illustrates the appearance of the respective two isomers of both mono- and di-CPANs during the chlorination process of Phe.

In the structural investigation of the chlorinated DBPs of Phe, successive examinations were conducted on reaction systems with varying molar ratios of Phe to available chlorine (Phe:HOCl). It was found that the product chlorobenzene (P-114) only appeared in systems with available chlorine concentrations at or above twice the molar ratio, while more toxic CPANs continued to be produced at lower available chlorine concentrations (Table 3).

#### 2.1.3. DBPs of Trp Chlorination

**Figure 3:**
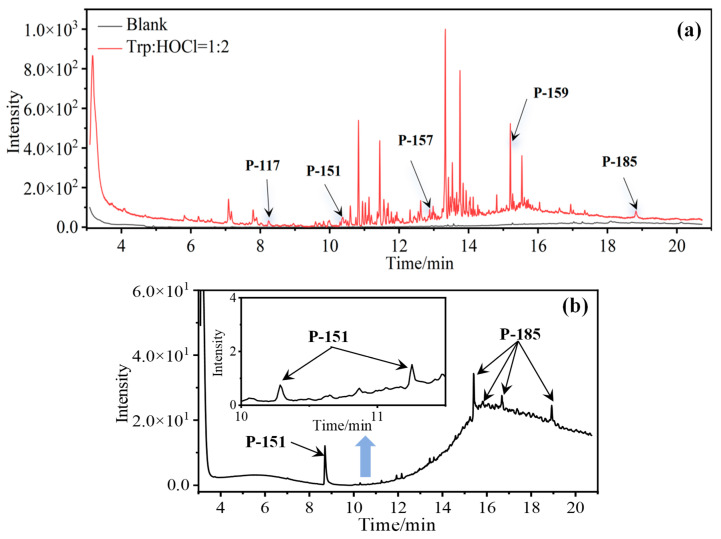
(**a**) Total ion chromatogram of Trp chlorination (pH = 7.0, 25 °C, Phe:HOCl = 1:2, 24 h) and (**b**) Selective ion monitoring chromatogram of generated CPANs under the same conditions.

In the case of Trp, a total of five DBPs were identified during the chlorination process. Detailed chromatographic representations of these DBPs are displayed in Figure 3, with specific product information provided in Table 1. The unique benzene-fused heterocyclic structure of Trp resulted in the formation of 3-quinolinecarboxaldehyde (P-157) and indole-3-acetaldehyde (P-159). Similar to the observations with Tyr and Phe, chlorination of Trp also engendered the generation of CPANs, signifying the susceptibility of the five-membered ring in Trp to the ring-opening process. Moreover, during Trp chlorination, three isomers of mono-CPAN (P-151) can be generated, while four isomers of di-CPAN are formed (Figure 3b).

The reaction results of Trp with chlorine at different molar ratios (Trp:HOCl) are collected in Table 4. It is observed that when the concentration of available chlorine is sufficiently excessive, the formation of chlorinated DBPs (P-151 and P-185) is maximized. For instance, P-157 is only produced at Trp:HOCl = 1:2 or even lower ratios. This trend mirrors the pattern observed in the formation of DBPs from the preceding two AAs.

As evident from Table 1, a total of 15 byproducts are identified during the chlorination of three aromatic AAs, predominantly comprising various aldehydes and acetonitriles containing aromatic rings, as well as phenols and halobenzenes. Notably, CPANs, which are highly toxic aromatic DBPs, are detected in the chlorination processes of all three aromatic AAs. The observed CPANs include multiple monochlorinated and dichlorinated isomers. This demonstrates that Tyr, Phe, and Trp may serve as precursors for CPANs in aquatic environments. Moreover, the residues of three aromatic AAs are undetectable after 24 h of reaction, indicating their complete conversion during the chlorination process.

### 2.2. Kinetics of Chlorination for Three Aromatic AAs

#### 2.2.1. Kinetics of Chlorination Reactions at Various Available Chlorine Concentrations

**Figure 4:**
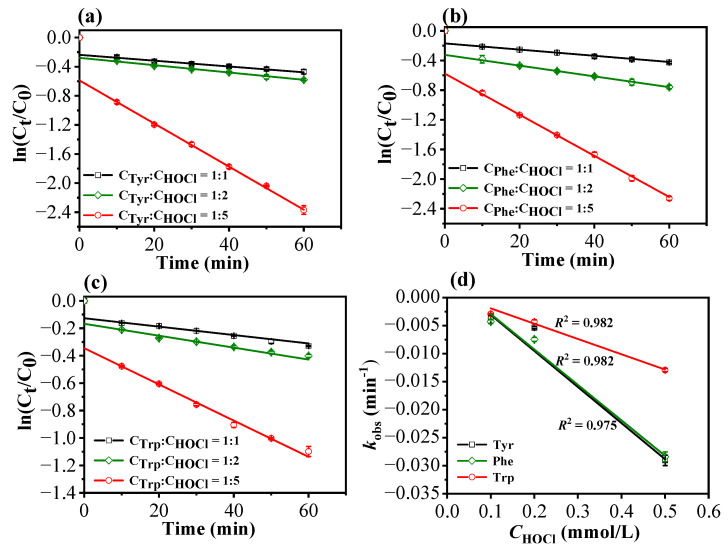
Reaction kinetics of Tyr (**a**), Phe (**b**), and Trp (**c**) at different ratios of available chlorine concentration (pH = 7.0, 25 °C). (**d**) Effects of active chlorine on *k*_obs_ of the three AAs.

Figure 4 illustrates the chlorination (or degradation) kinetics of Tyr, Phe, and Trp across varied available chlorine concentrations. Given the rapidity of chlorination reactions, samples were extracted at 10 min intervals over a 1 h chlorination duration. Despite the potential model intercept, the fitting curves demonstrate strong correlations, with *R*^2^ values exceeding 0.98. This result suggests that the chlorination reaction is first-order toward the three AAs under the specified conditions of active chlorine concentration and reaction time. Furthermore, the observed rate constants (*k*_obs_) exhibit linear correlations with active chlorine concentration (Figure 4d), implying that the reaction is also first-order with respect to active chlorine. Consequently, the chlorination of the three AAs is expected to follow second-order reaction kinetics. In response to the presence of the model intercept for t = 0, which shows potential deviations from zero, we propose that there may be a very rapid initial reaction for each AA, likely involving the Cl-substitution on α-amino group [34], followed by subsequent slow and measurable reaction steps during the chlorination process. It can also be observed from Figure 4 that increasing the chlorine dosage significantly promotes the degradation rate for all three AAs. Notably, at a chlorine concentration ratio of 1:5, degradation rates substantially surpass those observed at a ratio of 1:2.

Table 5 presents the kinetic coefficients relevant to the chlorination of Tyr, Phe, and Trp. Analysis of the data reveal that chlorination rates of the three AAs follow the order of Phe > Tyr > Trp at a C_AA_:C_HOCl_ ratio of 1:1 and 1:2, while the chlorination rate of Tyr exceeds that of Phe and Trp when the C_AA_:C_HOCl_ ratio is 1:5. Notably, the degradation rate associated with Trp is lower than those of Tyr and Phe across all available chlorine concentrations. This discrepancy suggests that the chlorination kinetics of Trp are comparatively less influenced by fluctuations in available chlorine concentration, attributable to inherent structural differences between these AAs. The specific benzene-fused heterocyclic structure inherent to Trp likely underpins its distinct chlorination kinetics compared to the structurally analogous Tyr and Phe.

#### 2.2.2. Kinetics of Chlorination Reactions at Various pH Levels

**Figure 5:**
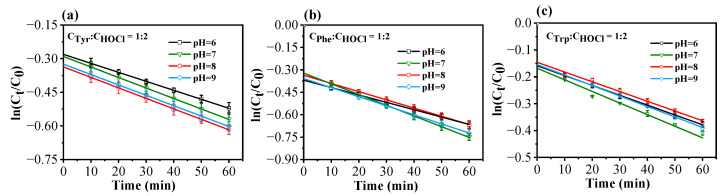
Chlorination reaction kinetics of Tyr (**a**), Phe (**b**), and Trp (**c**) at different pH values (C_AA_:C_HOCl_ = 1:2, 25 °C).

Figure 5 displays the chlorination kinetics of three aromatic AAs at differing pH levels (the reaction concentration is selected as C_AA_:C_HOCl_ = 1:2). Given the expeditious nature of chlorination reactions, sampling intervals were also set at 10 min increments, with the chlorination reaction duration maintained at 1 h. It can be observed that pH variations potentially influence the reaction rates under consistent conditions.

Table 6 provides an overview of the kinetic coefficients for chlorination reactions involving Tyr, Phe, and Trp across varying pH levels. The data presented in Figure 5 and Table 6 collectively show that the three AAs have the fastest reaction rate at pH = 7.0, which decreases when the pH increases to 8.0 and decreases further for Tyr as the pH increases to 9.0. In contrast, the chlorination rates of Phe and Trp at pH = 9.0 increase compared to those at pH = 8.0. Moreover, the chlorination rates of the three AAs under alkaline conditions (pH = 8.0 and 9.0) are greater than those under a pH value of 6.0, suggesting a faster chlorination reaction of these three AAs under neutral and weak alkaline conditions. Remarkably, the chlorination rate of Phe is the most susceptible to pH fluctuations among the three AAs.

The structural specificity of the aromatic ring moieties in the three AAs contributes to their disparate reaction rates and preferences, with the overall order of degradation rates being Phe > Tyr > Trp. AAs characterized by accelerated reaction rates may yield a greater abundance of DBPs within a given timeframe, necessitating heightened attention. To effectively manage DBP formation, a judicious selection of disinfection conditions based on the preferred disinfection pH value for each amino acid type is warranted.

## 3. Materials and Methods

### 3.1. Chemicals

Sodium hypochlorite (available chlorine ≥ 5%), formic acid, acetonitrile, ascorbic acid, and methyl tert-butyl ether were purchased from Aladdin Biochemical Technology Co, Ltd. (Shanghai, China). Tyr, Phe, and Trp were sourced from D&B Biotechnology Co., Ltd. (Shanghai, China). All chemicals used in this study were of analytical grade, and ultrapure water from the Milli-Q Progard water purification system was used.

### 3.2. Experimental Procedures

#### 3.2.1. Experiments on Chlorination of Tyr, Phe, and Trp

The precise volumes of each AA and freshly prepared chlorine reserve solution were separately pipetted, with the AA concentration set at 0.1 mmol/L. Molar concentration ratios of each AA to available chlorine were rigorously controlled to be 2:1, 1:1, 1:2, 1:5, and 1:10, respectively. AAs, disinfectant, and phosphate-buffered saline (PBS) were added sequentially to 100 mL brown volumetric flasks, ensuring the maintenance of a pH value of 7.0 throughout the reaction, with final volume adjustments made using purified water. To facilitate sufficient reaction between chlorine and AAs, samples were incubated in a light-protected constant-temperature incubator at 25 °C for 24 h. Upon completion of the reaction period, appropriate quantities of ascorbic acid were introduced to neutralize residual chlorine and terminate the chlorination process.

A 30 mL aliquot of the sample underwent sequential addition of approximately 10 mL of extractant methyl tert-butyl ether three times, with vigorous shaking (2500 rpm for 5 min) following each addition. After three repetitions of extraction, the supernatant was transferred to a 10 mL vial. Then, 8 g of anhydrous sodium sulfate was added, allowing the mixture to stand for 60 min. Subsequent enrichment and concentration to 1 mL were achieved via nitrogen blowing, followed by filtration through a 0.22 μm organic filtration membrane. The filtered samples were then subjected to analysis using gas chromatography-mass spectrometry (GC-MS, 7890B-5977, Agilent Technologies, Santa Clara, CA, USA).

#### 3.2.2. Kinetics of the Chlorination Reaction of Tyr, Phe, and Trp

To explore the potential effects of chlorine concentrations on chlorination reaction kinetics of three AAs, the disinfectant sodium hypochlorite was reacted with each AA solution at varying molar ratios (C_AA_:C_HOCl_ = 1:1, 1:2, and 1:5). The solution pH value was adjusted to 7.0 using PBS, and reaction flasks were kept in darkness at 25 °C for 10–60 min. Reaction termination occurred via the addition of ascorbic acid solution following the designated reaction time.

To further evaluate the impact of pH values on chlorination kinetics, the reaction was conducted by adjusting the solution pH values to 6.0, 7.0, 8.0, and 9.0. Maintaining a constant concentration ratio of C_AA_ to C_HOCl_ at 1:2, the reaction mixture underwent incubation at 25 °C within a light-protected constant-temperature incubator for a duration spanning 10 to 60 min. Termination of the reaction was achieved by introducing an ascorbic acid solution.

### 3.3. Instrument Conditions and Analysis Methods

To identify the potential DBPs, the GC-MS analysis utilized an HP-5ms column (J&W HP-5ms, 30 m × 0.25 mm, 0.25 μm, Agilent Technologies, Santa Clara, CA, USA) with high-purity helium as the carrier gas. The flow rate of carrier gas was 3 mL/min, and a sample volume of 1 μL was injected. The programmed column temperature consisted of an initial hold at 50 °C for 3 min, followed by ramping to 110 °C at 25 °C/min for 3 min, and finally to 220 °C at 15 °C/min for 5 min. The total runtime was 22 min, with an inlet temperature set at 220 °C. Electron ionization (EI) at 70 eV was employed, with ion source and interface temperature set at 230 °C and 220 °C, respectively. Both full-range (*m/z* 50~500) and selected ion monitoring modes (*m/z* 89, 114, 116, 150, 151, and 185) were utilized for mass spectrometric analysis, with a solvent delay of 3 min.

The concentration of AAs after the chlorination reaction was quantified using High-Performance Liquid Chromatography (HPLC, 1260 Infinity II, Agilent Technologies, Santa Clara, CA, USA) equipped with an EC-C18 column (Poroshell 120 EC-C18, 3.0 × 100 mm, 2.7 μm, Agilent Technologies, Santa Clara, CA, USA) and mobile phases comprising 0.1% formic acid water (A) and acetonitrile (B). The running time was set at 4 min, with detection parameters tailored to the specific AAs (Table 7).

## 4. Conclusions

In this study, the simulation of the chlorination process targeting three aromatic AAs (Tyr, Phe, and Trp) resulted in the identification of 15 DBPs, primarily consisting of various aldehydes, phenols, nitriles, and other chlorinated compounds. This comprehensive elucidation significantly enriches the existing DBP database. Of particular concern is the detection of highly toxic aromatic DBPs known as CPANs, including multiple monochlorinated and dichlorinated homologs. This highlights the potential of Tyr, Phe, and Trp as precursors for CPANs in aquatic environments, posing challenges for their removal in conventional water treatment processes. Consequently, eutrophic waters characterized by elevated levels of aromatic AAs demand heightened scrutiny during chlorination procedures. Distinct variations in relative chlorination rates among the three AAs were also observed, with Phe exhibiting the highest reactivity, followed by Tyr and Trp, at both the C_AA_:C_HOCl_ ratio of within 1:2 and different pH values ranging from 6.0 to 9.0. Furthermore, faster chlorination reaction of these three AAs can be anticipated under neutral and even weak alkaline conditions. Tailoring disinfection processes to AA attributes is essential, considering their diverse functional group structures. AAs with accelerated reaction rates may yield more byproducts, necessitating enhanced vigilance in water treatment protocols.

## Figures and Tables

**Table 1:** Information on DBPs formed from chlorination of three aromatic AAs.

Source of DBPs	Compound	Proposed Structure	Characteristic Ion	Match
Chlorination DBPs of Tyr	P-108	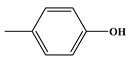	51, 77, 108	88.6%
	P-176	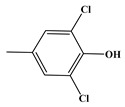	77, 141, 176	92.0%
	P-136	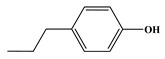	77, 107, 136	86.7%
	P-142	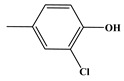	77, 107, 142	91.4%
	P-133	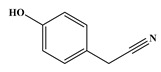	78, 106, 133	90.4%
	P-190	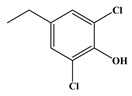	77, 155, 190	89.1%
Chlorination DBPs of Phe	P-120	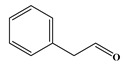	65, 91, 120	95.5%
	P-106	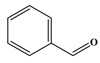	51, 77, 106	96.7%
	P-114	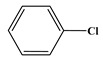	77, 112, 114	82.4%
	P-126	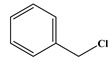	91, 126	92.5%
Chlorination DBPs of Trp	P-157	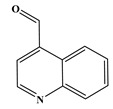	75, 129, 157	93.1%
	P-159	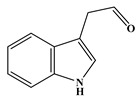	103, 130, 159	90.8%
Common DBPs of three aromatic AAs	P-117	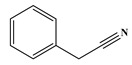	90, 117	92.5%
	P-151	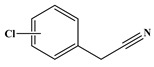	89, 116, 151	87.9%
	P-185	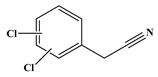	89, 150, 185	87.6%

**Table 2:** Distribution of DBPs of Tyr chlorination under different ratios of chlorine concentration (pH = 7.0, 25 °C, 24 h). √ denotes the presence of the respective product in each reaction system.

Tyr:HOCl	P-136	P-108	P-142	P-190	P-176	P-117	P-133	P-151	P-185
2:1						√		√	
1:1	√		√			√	√	√	√
1:2	√	√	√	√	√	√	√	√	√
1:5	√	√	√	√	√	√	√	√	√
1:10	√	√	√	√	√	√	√	√	√

**Table 3:** Distribution of DBPs of Phe chlorination under different ratios of chlorine concentration (pH = 7.0, 25 °C, 24 h). √ denotes the presence of the respective product in each reaction system.

Phe:HOCl	P-106	P-120	P-114	P-126	P-117	P-151	P-185
2:1					√	√	
1:1	√	√		√	√	√	√
1:2	√	√	√	√	√	√	√
1:5	√	√	√	√	√	√	√
1:10	√	√	√	√	√	√	√

**Table 4:** Distribution of DBPs of Trp chlorination under different ratios of chlorine concentration (pH = 7.0, 25 °C, 24 h). √ denotes the presence of the respective product in each reaction system.

Trp:HOCl	P-157	P-159	P-117	P-151	P-185
2:1		√		√	
1:1		√	√	√	√
1:2	√	√	√	√	√
1:5	√	√	√	√	√
1:10	√	√	√	√	√

**Table 5:** Kinetic coefficients for the chlorination reactions of Tyr, Phe, and Trp at different ratios of available chlorine concentration.

Chlorination	Tyr		Phe		Trp	
	*R* ^2^	*k*_obs_ (min^−1^)	*R* ^2^	*k*_obs_ (min^−1^)	*R* ^2^	*k*_obs_ (min^−1^)
C_AA_:C_HOCl_ = 1:1	0.987	−3.655 × 10^−3^	0.997	−4.254 × 10^−3^	0.999	−2.901 × 10^−3^
C_AA_:C_HOCl_ = 1:2	0.994	−5.412 × 10^−3^	0.998	−7.373 × 10^−3^	0.989	−4.540 × 10^−3^
C_AA_:C_HOCl_ = 1:5	0.999	−2.986 × 10^−2^	0.998	−2.809 × 10^−2^	0.996	−1.333 × 10^−2^

**Table 6:** Kinetic coefficients for the chlorination reactions of Tyr, Phe, and Trp at different pH values.

Chlorination C_AA_:C_HOCl_ = 1:2	Tyr		Phe		Trp	
	*R* ^2^	*k*_obs_ (min^−1^)	*R* ^2^	*k*_obs_ (min^−1^)	*R* ^2^	*k*_obs_ (min^−1^)
pH = 6.0	0.998	−4.089 × 10^−3^	0.997	−4.841 × 10^−3^	0.998	−3.714 × 10^−3^
pH = 7.0	0.994	−4.767 × 10^−3^	0.999	−7.094 × 10^−3^	0.979	−4.448 × 10^−3^
pH = 8.0	0.998	−4.590 × 10^−3^	0.998	−5.539 × 10^−3^	0.989	−3.874 × 10^−3^
pH = 9.0	0.997	−4.550 × 10^−3^	0.998	−5.985 × 10^−3^	0.996	−3.989 × 10^−3^

**Table 7:** Conditions for liquid-phase detection of three AAs.

AAs	Flow Rate	Injection Volume	Elution Gradient	Excitation Wavelength	Temperature
Tyr	0.4 mL/min	30 μL	4% B, 96% A, 0–4 min	280 nm	20 °C
Phe	0.6 mL/min	30 μL	6% B, 94% A, 0–4 min	210 nm	35 °C
Trp	0.6 mL/min	30 μL	10% B, 90% A, 0–4 min	230 nm	35 °C

## Data Availability

Data are contained within the article.
